# Exploring Clients’ Experiences of Transitioning Mental Health Nursing Care from an In-Person to a Virtual Format due to the COVID-19 Pandemic

**DOI:** 10.1177/08445621231221033

**Published:** 2023-12-13

**Authors:** Jean-Laurent Domingue, Lisa Murata, Chijindu Ukagwu, Billie Pryer, Shruti Patel, Jennifer Neves, Tariq Iqbal

**Affiliations:** 1 6363 University of Ottawa, Ottawa, ON, Canada; 2 97559 Royal Ottawa Health Care Group, Ottawa, ON, Canada

**Keywords:** COVID-19, mental health nursing, phenomenology, qualitative methods, transitions, virtual care

## Abstract

The onset of the COVID-19 pandemic led mental health professionals to change the way they engaged with clients, often replacing in-person consultations with virtual ones via telephone or videoconferencing. While studies have investigated the delivery of virtual physical health care, only a handful have investigated the delivery of virtual mental health. These specifically focussed on the outcomes of virtual care whether experiential, practical, or empirical. The transition from in-person to virtual care delivery due to the COVID-19 pandemic has been unexplored. Accordingly, the purpose of the study was to: (1) Explore the experiences of clients who had to transition from an in-person to a virtual provision of mental health care due to the COVID-19 pandemic, and; (2) Explore the nurses’ experiences of this technological transition. Using an interpretive phenomenology methodology, semi-structured interviews were conducted with nurses and clients who have experienced the in-person to virtual transition of service delivery at a tertiary mental health hospital in Ontario, Canada. In this article, we focus on the results stemming from our interviews with clients. The themes generated from the analysis of client experiences are 1) the psychosocial impact of the COVID-19 pandemic on clients, (2) mixed feelings of clients towards nursing care delivered via technological means and (3) the role of nurses regarding transitioning of in-person care to technology-mediated care. These findings are relevant as mental health care hospitals are considering how they will deliver services once concerns with the transmission of the COVID-19 virus are resolved.

## Introduction

In early 2020, the COVID-19 pandemic brought the world to its knees. It forced us to rethink and readjust in all aspects of life; personal, professional, leisure, etc. Beyond the practical inconveniences of how these new ways of doing engendered, they caused for rates of mental health problems to soar, especially for those whose health and employment were affected by COVID-19 or by the governmental measures put in place to stop the spread of the virus (Mental Health America (MHA), 2021; Pongou et al., 2022; Statistics Canada, 2021). In Canada, individuals felt increased psychological distress, depression and anxiety (Pongou et al., 2022; Statistics Canada, 2021) – and mental health professionals responded. However, mental health professionals had to change the way they engaged with clients, often replacing in-person consultations to take place on virtual platforms.

A variety of studies have investigated the delivery of virtual mental health care (Adams et al., 2018; Sugarman et al., 2021; Uscher-Pines et al., 2020). However, the focus of these studies revolves around the clinical outcomes of virtual care (See, for example, Brenes et al., 2012; Pratt et al., 2015; Talarico, 2021). The transition from in-person to virtual care delivery, from the moment in-person services cease to the moment recipients and providers are fully accustomed to the new virtual way of doing, has been left virtually unexplored. Further, the studies conducted generally grouped mental health care professionals in one homogenous group without specific regard to their professional designations. By doing so, they may have silenced peculiarities related to individual professions, such as occupational therapists, psychiatrists, or nurses. For mental health nurses, understanding the experiences of transitioning in-person to virtual care is particularly important to explore because a central tenet of mental health nursing rests on the development of therapeutic interpersonal relationships (Eckroth-Bucher, 2001; Laskowski, 2001; Peplau, 1952; Travelbee, 1971).

The purpose of the study we conducted was thus to fill this gap and better understand how clients living with mental illnesses (CLWMI) and mental health nurses experienced the transition of mental health nursing services from in-person modes of delivery to virtual modes due to the COVID-19 pandemic. In this article, we focus on our findings related to the experiences of CLWMI.

## Background

In the field of mental health and psychiatry, telehealth has proved useful across the care spectrum, from psychiatric assessments to diagnosis and treatment, crisis evaluation, consultations, symptom monitoring, and post-discharge support (Farrar, 2015). Specifically, evidence from numerous studies support tele mental health (TMH) nursing practice for a variety of psychiatric conditions (e.g., depression, bipolar disorder, autism spectrum disorder) and it has been demonstrated to be effective across the life span from pediatric to geriatric populations (Farrar, 2015).

The TMH technology allows nurses to provide services in areas where there is a shortage of mental health specialists. It permits nurses to increase their patient load through a more efficient workflow and improve patient quality of life while continuing to provide safe, quality care to their patients (Farrar, 2015). Adams et al. (2018) found that TMH services consistently proved equal to or superior to in-person care in all age groups, but they underline the possibility that some populations may not lend themselves well to this modality (e.g., persons with severe schizophrenia or cognitive/sensory impairments).

Clients who receive TMH care report a high satisfaction with this service (Bhandari et al., 2011; Ellington, 2013; Naik et al., 2021). They find TMH services to be responsive to their needs that they would use these services again, and that they would recommend them to other people (Saurman et al., 2011). In fact, although staff were concerned that patients may not be comfortable communicating with a mental health practitioner using telehealth technology, patients reported feeling very comfortable with the experience (Bhandari et al., 2011).

A review of the literature identified many benefits of TMH care. Among other things, TMH increased accessibility to mental health services, increased medication adherence, had a limited impact on therapeutic engagement, and allowed for the effective management of mental health symptoms.

### Mental health care accessibility

Virtual delivery of mental health services reduces barriers for many populations, such as those who need to travel long distances, who take public transit or rely on someone else for transport, with mobility issues, medical concerns, those living remotely, and those with work/childcare obligations (Sugarman et al., 2021; Wynn & Sherrod, 2012). The use of TMH also improves accessibility for individuals with increased vulnerabilities related to their psychiatric condition like social anxiety (Sugarman et al., 2021; Uscher-Pines et al., 2020).

The use of TMH services has been particularly useful for hospitals and clinics in rural settings. For example, Southard et al. (2014) highlight partnerships between rural hospitals and urban mental health centers allowed for increased accessibility to this specialty in emergency departments (ED) and acted as a safety net for clinicians. In Trondsen et al. (2014), mental health nurses in a regional psychiatric center expressed feeling less uncertain when confronted with psychiatric emergencies knowing that a specialist was a video call away. Relatedly, Bhandari et al. (2011) found that, in some rural hospitals, such partnerships reduced by 100 percent the need to hold unstable patients with mental health disorders, thereby allowing for patients to be treated and discharged directly from the emergency department without being admitted to EDs or mental health units.

### Medication adherence

The TMH services can also increase patient adherence to treatments. Using a telephone intervention with patients living with schizophrenia, Uslu and Buldukoglu (2020) found increases in both the insight into the necessity of taking medications and adherence to medications. Montes et al. (2010) had similar results, identifying that TMH services increased medication adherence in persons with schizophrenia. They also found that adherence to treatment increased progressively after each phone call.

However, not all populations benefit equally from TMH services. In Talarico (2021), while adherence to medication treatment was found to be increased by TMH services (82% by day 30 and 77.5% by day 60), medication adherence decreased for patients with dual diagnoses despite a TMH intervention (66.2% at 30 days and 33% by 60 days). Relatedly, Alston et al. (2019) underscored that youths experiencing first episode psychosis were more likely to disengage from services with an entirely virtual approach as opposed to an in-person approach. They suggested this population may benefit from a hybrid model that combined virtual and in-person services.

### Therapeutic engagement

Establishing a therapeutic relationship is an essential component of mental health nursing practice (Eckroth-Bucher, 2001; Laskowski, 2001; Peplau, 1952; Travelbee, 1971). Several studies discussed the impact of establishing the therapeutic relationship in virtual care. Evans et al. (2017) demonstrated that therapeutic alliance could be established with virtual nurse-patient interventions, putting forward as evidence their study's high retention rate: they lost only 10 participants in their original sample of 695 pregnant women diagnosed with depression. However, Trondsen et al. (2014) found that patient engagement in care was influenced by the way TMH is deployed: videoconferences involving multiple members of the team appeared to facilitate stronger patient involvement when compared to a one-on-one telephone consultation with a nurse.

Recipients of care did not have a homogenous experience. Johnson (2020) found that youths with low-self esteem, body image issues, or psychological issues like social anxiety, many of whom are already spending their days on a computer, found it difficult to engage virtually. Turner et al. (2018) reported that some practitioners felt like a disembodied voice and that some therapies delivered over the phone felt hurried suggesting length of interaction may compromise aspects of establishing the therapeutic alliance. Similarly, Uscher-Pines et al. (2020) reported that several providers found it more challenging to make meaningful connections with patients through TMH services because visits tended to be shorter and conversations shallower. In Sugarman et al. (2021), although clinicians expressed that TMH services gave them the opportunity to build rapport with patients by allowing them to observe family dynamics in their intimacy and making patients more at ease because they are at home, they acknowledged that in-person care provision may be more appropriate for group therapy, family therapy, and initial assessment visits.

### Mental health symptom management

The use of TMH interventions has been proven useful in managing a variety of psychiatric symptoms. Utilizing an automated telehealth intervention strategy, Pratt et al. (2015) noted significant decreases in psychotic symptom severity. This study also found improvement in depressive symptoms, ability to perform general health behaviors, and subjective mental health well-being. Montes et al. (2010) demonstrated that their telephone intervention group showed significant improvement in positive, depressive, cognitive, and global symptoms when compared to their control group. This trend holds across other populations with different diagnoses. Brenes et al. (2012) conducted a study enrolling adults over sixty living with a diagnosis of general anxiety disorder. They found that participants who received telephone-delivered cognitive behavioral therapy (CBT) had significant declines in general anxiety, worry, anxiety sensitivity, and insomnia when compared to the usual treatment group who received a brochure on anxiety and information on referrals.

In brief, the scientific literature highlights many benefits of TMH services, from increasing medication adherence to improving symptoms of psychiatric illnesses. It also illustrates that clients living with mental illnesses are satisfied with the professional services they receive via virtual modes of communication. However, the experiences of clients and providers during *a period of transition from in-person to virtual care delivery* has been left virtually unexplored. Further, the scientific literature on TMH services generally groups mental health care professionals in one homogenous group without specific regard to their professional designations. By doing so, they may silence peculiarities related to individual professional disciplines, such as occupational therapy, psychiatry, or nursing. Thus, it is the period of transition between in-person mental health services to virtual mental health services and the discipline of nursing, and its practices, that were the matter of investigation in this study.

## Theoretical influences

The concept of transition is central to the research problem investigated. The COVID-19 pandemic has caused patients and nurses alike to undergo a plethora of transitions. While these transitions have caused for some to develop new ways of living, for others these transitions equated to loneliness, anxiety, and uncertainty.

Ibrahim Meleis, a prominent nursing scholar, developed a middle-range theory about the concept of transitions in nursing in the late 1990s/early 2000s (Meleis et al., 2000). Acknowledging that transitions are diverse and infused with complexity, with the help of her colleagues, she wrote: “clients’ daily lives, environments, and interactions are shaped by the nature, conditions, meanings, and processes of transition experiences” (Meleis et al., 2000, pp. 12–3). Meleis and colleagues (2000) explained that

“nurses often are the primary caregivers of clients and their families who are undergoing transition. [Nurses] attend to the changes and demands that transitions bring into the daily lives of clients and their families [by] facilitate[ing] the process of learning new skills related to clients’ health and illness experiences” (p.13).

The theoretical framework they proposed involves four interconnected components: nature of transitions (types, patterns, and properties of transition experiences), transition conditions (facilitators and inhibitors), pattern of response (process and outcome indicators), and nursing therapeutics (Meleis et al., 2000). According to this framework, transitions have various origins, frequencies, patterns of appearance, and everyone experiences transitions in their own way. The environment in which individuals undergo transitions and the personal characteristics of each individual (culture, socio-economic status, etc.) facilitates and/or impedes their response to them, and their overall experience in relation to them. As caregivers closest to clients when health-related transitions are experienced, nurses are well positioned to intervene therapeutically and assist clients in achieving healthy transition outcomes (Meleis et al., 2000).

Meleis's transition framework allows for the COVID-19 pandemic to be conceptualized as a situational public health event that caused transitions of all sorts. Individuals, each with their own personal characteristics and living in their own environments (social, physical, economic, cultural, etc.), experienced and adapted to these transitions in their own unique way. Indeed, clients and nurses who experienced the transition from in-person to virtual provision/reception of mental health care may have been influenced by many factors ranging from their technological knowledge to their access to the internet and their ability to concentrate. Exploring the unique experiences of clients and nurses and the factors that contributed or impeded a successful transition to virtual care was the focus of our study.

## Methodology

Given the exploratory nature of the research objectives and the importance of human experiences in the concept of transition, a qualitative methodology, namely interpretative phenomenology, was chosen. Phenomenology is a methodology concerned with the way individuals perceive and experience the world in which they live. Husserl is said to be the founder of phenomenology (Rodriguez & Smith, 2018). Rooted in positivist tradition, he was interested in uncovering the essence of human experience. Building on Husserl's thought, later theorists, such as Heidegger, moved away from an objective epistemological dimension of phenomenology and introduced interpretive dimensions (Finlay, 2009). Descriptive accounts of experiences were replaced by interpretive accounts; the purpose of inquiry was no longer related to the universal consciousness of the human mind, but it rather stemmed from the meaning individuals made of their lives and the various phenomena they experienced (Finlay, 2009). Interpretive phenomenology allows “researchers to investigate how individuals make sense of their experiences. It is assumed that people are ‘self-interpreting beings’, which suggests that they are actively engaged in interpreting the events, objects, and people in their lives” (Pietkiewicz & Smith, 2014, p. 8). Indeed, interpretive phenomenologists are interested by the way individuals interact with the world; that is how they relate to other individuals, space, and time (Finlay, 2009).

Interpretive phenomenology aligned well to the research objectives because it allowed for the inquiry to be focused on the sense CLWMI and mental health nurses gave to their experience of transitioning mental health care from in-person to virtual modes of delivery. Specifically, notions of time, space, and interpersonal relationships in the context of such a transition were explored during semi-structured interviews. To structure the interviews, a phenomenological interview guide, developed based on Meleis et al. (2000) theoretical model and on empirical literature about the provision of virtual mental health care was used.

Six CLWMI and six mental health nurses were recruited for this phenomenological study. Participation consisted of one meeting with a member of the research team lasting approximately 60 minutes. The meeting took place virtually via the Zoom videoconferencing platform and in person (depending on the preference of the participant and COVID-19 governmental restrictions). All but one participant consented to the audio recording of their interview. For the participant who was opposed to it (Client 6), the interviewer took notes to document the participant's answers.

### Recruitment of CLWMI

Nurses at an urban multi-site psychiatric hospital were asked to obtain their clients’ permission to be contacted by a member of the research team. With the clients’ permission, nurses provided the research team with their names and contact information. The first author was then able to contact prospective participants and recruit them for the study. Inclusion criteria for clients were: (1) Having experienced the transition of in-person to virtual provision of nursing care due to the COVID-19 pandemic; (2) Being ≥16 years of age; (3) Understand and speak French or English; and (4) Capable to comprehend and provide consent to the study. There were no criteria for excluding a client from this study.

#### Sample of CLWMI

The research team attempted to recruit the 14 CLWMI whose names and contact information were obtained through the permission to contact process described above. Of these, six CLWMI accepted to participate, five declined and two did not return communications. The six participants who were recruited all lived with schizophrenia or a psychotic spectrum disorder. Table 1 provides the demographic profile of the study participants.

**Table 1.** Demographic profile of clients living with mental illness.

Client	Age	Ethnicity	Gender Identity	Education	Years Receiving Mental Health Care
Client 1	47	European	Man	Grade 8	10
Client 2	45	North American with Indigenous Roots	Man	BA	20
Client 3	60	European	Woman	Professional	34
Client 4	63	Czech	Woman	BA	38
Client 5	60	French	Woman	Grade 12	42
Client 6	30	Caucasian and Jamaican	Man	Grade 12	7

### Recruitment of mental health nurses

Recruitment of mental health nurses was achieved through an invitation which was distributed to all nurses via the hospital's internal e-mailing system. Inclusion criteria for mental health nurses were: (1) Having experienced the transition from an in-person to virtual delivery of nursing mental health care due to the COVID-19 pandemic; (2) Understand and speak French or English. There were no criteria for excluding mental health nurses from this study.

### Data analysis

Data analysis took place in a three-stepped approach (Creswell & Poth, 2018). Although presented successively, these steps were not completed in a predetermined approach, but were rather iterative. The steps followed were (1) multiple reading of the transcripts and making notes, (2) transform notes into emergent themes, (3) seeking relationships and clustering themes. The purpose of these steps was to produce a description of the lived experience of clients and nurses regarding the transition to virtual delivery of mental health care due to the COVID-19 pandemic and to understand the meaning they give to their experience of this phenomenon. We used the NVivo data management software to assist in this process. For this article, we focus our findings related to the analysis of the experiences of CLWMI. Mental health nurses’ experiences will be discussed elsewhere.

### Ethical considerations

Research ethics board approval was obtained from the institution where participants were recruited (#2021032) and from the first author's institution (H-11-21-7622). Informed consents were obtained from participants before interviews were conducted.

### Rigour

Many scholars have proposed rigour criteria to produce (and evaluate) quality and robust qualitative studies (see for example, Davies & Dodd, 2002; Tracy, 2010). Tracy (2010) suggested that “each criterion of quality can be approached via a variety of paths and crafts, the combination of which depends on the specific researcher, context, theoretical affiliation, and project” (p. 837). For the present study, the credibility criterion was of particular importance. Credibility refers to the ability to justify study findings based on the data; “showing rather than telling” (Tracy, 2010, p. 840). Credibility was ensured through the consistent use of interview excerpts throughout the results section to justify the various study findings. Further, in their “Standards for Assessing the Quality of a Phenomenology” (p. 273), Creswell and Poth (2018) propose that the use of a clear procedure to analyze phenomenological data is needed for a phenomenology to be rigorous. In the present study, this criterion was ensured with the use of Creswell and Poth's three-stepped approach for data analysis and with a clear description and application of a theoretical framework to make sense of the data.

## Findings

Interviews with CLWMI generated rich data that we organized in three overarching themes: (1) Psychosocial impact of COVID-19 on CLWMI, (2) Mixed feelings of CLWMI towards nursing care delivered via technological means and (3) The role of nurses regarding transitioning in-person care to technology-mediated care. These results illustrate, on the one hand, the profound impact COVID-19 had on the lives of CLWMI and, on the other, the importance of technology-mediated care for CLWMI to maintain a semblance of normality, in both the social and therapeutic domains. Further, the results highlighted the central role of mental health nurses in supporting CLWMI through the early global response to the COVID-19 pandemic and to the transitioning of mental health care from an in-person to a technology-mediated mode of delivery.

### Psychosocial impact of COVID-19 on CLWMI

On a psychosocial level, the COVID-19 pandemic was difficult for the participants. As social gatherings became restricted and public spaces, like hospitals and community centers, closed or reduced hours and access, they became increasingly isolated and lonely:

“People have become, they're getting used to being on their own. We used to get together once a month and do leisure outing and we would go to [government building], we go to the strawberry farm; we go to the beach we go to the museum; we go everywhere, once a month, and that was so nice” (Client 3).

The CLWMI interviewed viewed the hospital not only as a therapeutic space, in the medical sense, but also as a social space where they would meet friends, interact with people, and conduct leisure activities.

The therapeutic function of the psychiatric hospital also changed following the COVID-19 related lockdowns. Access to the hospital was restricted, which particularly affected clients who received outpatient treatment. Participants explained that the hospital initially stopped all in-person outpatient programs, which left them almost without mental health care:

“When the COVID-19 pandemic struck, all therapy-based groups (individual and group) stopped. The client explained that he would nevertheless receive phone calls from his nurse and his occupational therapist to see how he was doing. Phone calls persisted for two or three months until health care providers coordinated Zoom meetings” (Client 6).^1^

Despite the initial cancelation of formal individual-based and group-based therapies, mental health care professionals kept in contact with their clients via phone to help them navigate and cope with the COVID-19 related changes.

A couple of months after the initial widespread cancelations of in-person therapeutic services, the hospital set up virtual equivalents of these services via videoconferencing systems, like Zoom. While participants highlighted some limitations associated with videoconference-based mental health care (discussed elsewhere in the article), they were very grateful for having access to care in unprecedented times: “I just think it's a good option to have the Zoom. And it's definitely better than getting no care during this; it was really a saving thing, like it just saved us. I think it's progress. We gotta keep evolving” (Client 1). For this participant, videoconferencing was seen as progress and as a “saving” technology.

The introduction of videoconferencing-based care delivery also increased accessibility to various mental health services for certain individuals that did not have access to them before. The next excerpt highlights how videoconferencing-based care allowed for a CLWMI to access CBT and its related tools, flash cards in this instance:

“**Interviewer:** When COVID-19 hit and we had to change everything to virtual. How did that affect the recovery from your mental illness?

**CT5:** Well, I do have things to work on mentally. I do have that, it's not like it disappeared on me and I was…

**Interviewer:** Did it make it worse? Did it not change anything?

**CT5:** No, it changed some things because I didn't have my flash cards on my living room wall for nothing, right? Those are my tools.

**Interviewer:** So, before COVID you didn't have those tools on your wall?

**CT5:** No

**Interviewer:** So, have they helped you?

**CT5:** Yeah, well they helped me a bit to say that ‘holy shit I better do my flash cards if I want to feel better’” (Client 5).

While access to videoconferencing technologies acted for some CLWMI as somewhat of a “better than nothing” replacement for in-person mental health services, for others this transition to virtual care delivery opened new possibilities for treatment.

### Mixed feelings of CLWMI towards nursing care delivered via technology

Although participants expressed satisfaction about having access to videoconferencing technology in a COVID-19 context, they also had mixed feelings about this care-delivery medium for the future:

“I think it's a good option. I'm really into the option of having a choice. Maybe having it all virtual or all in person might not be the best idea in my opinion, especially when you have… even down to time. If you don't have the time to go all the way into the hospital on a certain day, it would help in that aspect also” (Client 1).

Participants considered videoconferencing technology to be a good option to have, but that it should not replace in-person services. To that effect, they highlighted key benefits and concerns associated with the use of videoconferencing to deliver mental health nursing care.

#### Benefits

Participants attributed three benefits to their experience of videoconference-based mental health nursing care: (a) connectedness during a pandemic, (b) convenience, and (c) improved technology literacy.

At a time where all in-person contacts were strictly limited, various technological means, including e-mail and videoconferencing, allowed for certain CLWMI to remain socially connected:

“The client mentioned that at the beginning of the COVID-19 pandemic, the hospital initiated an electronic ‘newsletter’ for the clients in his outpatient group. He found this helpful because it allowed him to stay connected with others despite not meeting in person. This newsletter contained recipes, inspirational mental health quotes, and many other things. He said he contributed to the newsletter by creating a music playlist with a friend. He said this was a fun project to do with his friends because it benefited other people” (Client 6).

Indeed, social connectedness via technology during the pandemic was mentioned by many of our participants as being an essential element for their mental wellbeing:

“If I had not been exposed to technology through [name of nurse]'s gentle encouraging, I wouldn't… I don't know what I would be today, I would be isolated in my apartment not knowing what to do. I can honestly say that I probably would be curled up in a ball and just sink. It wouldn't be the same” (Client 3).

Participants expressed that technology-mediated nursing care was a more convenient way to receive services than in-person care delivery. Among other things, participants highlighted that technology-mediated care reduced the time they spent preparing for in-person meetings and traveling to and from the hospital:

“I think it's more convenient. I don't have to take the bus to go to my groups anymore. I find it a very convenient way of being a part of a group and I get that social feeling where I feel like I'm connecting with other people, and I appreciate it” (Client 2).

For CLWMI who took medications with sedative effects, technology-mediated care delivery allowed them to attend therapeutic groups that take place early in the morning:

“When asked if the transition to virtual care brought benefits regarding travel time, he said it did not change anything because he lived in walking distance to the hospital. What he found more challenging was getting up in the morning for 09h00 groups at the hospital because of the medications he takes. He said that getting up and logging onto the computer was easier – and that he was less likely to arrive late (or miss) group when it was on Zoom” (Client 6).

Technology-mediated groups increased the accessibility of therapeutic groups for CLWMI who use medications with significant sedative effects. By eliminating the travel time between their house and the hospital, and the necessity to prepare themselves for in-person groups, CLWMI could wake up later and attend therapeutic groups delivered virtually.

The transition to technology-mediated nursing services forced CLWMI to learn technological skills they did not necessarily have previously:

“I was so scared. I was scared to ask people to show me how [to use technology] because I didn't want them to think that I was stupid because “Nothing? you don't know anything about technology?” and I said, “no. not a thing.” and they look at me like “where did you come from?”. So, it was really difficult in the beginning. Now I have people like my family members, they're proud of me because I've started to do that. It's a different world” (Client 3).

Learning technology was fear-inducing for certain CLWMI. This fear was associated with the negative perception others would make of the person's lack of technological literacy. Once CLWMI gained technological skills, their newly acquired skills become a source of pride. Some even transposed their newly acquired technological skills from the clinical space to the social space: “I was apprehensive, at first. I'm a little bit better with it now. Actually, yeah, I'm learning things from, like listening to songs on my iPad. I've been able to connect with somebody that's a singer and love him big time” (Client 5).

#### Concerns

Concerns expressed by participants associated with technology-mediated nursing care revolved around four themes: (a) accessibility, (b) anxiety, (c) privacy, and (d) social connectedness.

While participants highlighted the accessibility benefits of technology-mediated nursing care, they were worried that access to this technology may be a barrier for certain people. For example, Client 2 explicates that since the transition of care from in-person to virtual formats, they noticed that some of the CLWMI they had seen in groups held in-person were no longer present during virtual groups: “I know there's people who I know back before the pandemic who I don't see anymore. So unfortunately, they probably don't have the technology”. Similarly, Client 5's fear that certain people don’t have access to hardware necessary for technologically mediated nursing care, which limits their access to therapeutic groups: “I don't understand that people my age – like I'm still young, I'm still a young woman – but you know there are other people as well who need to have access to computers and to iPads or what not? Uh, tablets anything like that for them to be able to socialize online.” In other words, the accessibility benefits attributed to technologically mediated nursing care can only materialize if CLWMI have access to the hardware necessary to participate in virtual care, including computers, tablets, microphones, cameras, speakers, and an internet connection.

Participants expressed feeling initially very anxious when faced with new ways of accessing health care. Getting used to the novelty and unexpectedness of videoconferencing was the source of this anxiety:

“When nursing care was transferred to Zoom, the client said it was ‘new’ and ‘weird’. He explained that he found Zoom to be anxiety provoking because it was new, and he did not know what to expect. He mentioned that seeing everyone's face in a box was destabilizing” (Client 6).

For Client 6, the Zoom interface, which places the video feed of every person participating in the call in a gallery view (where you can see everyone at once), was highlighted as being an additional source of stress. Participants associated this anxiety with the technology-mediated omnipresent gaze from other group members they were being exposed to when participating in virtual group therapy.

Videoconferencing platforms, and the specific use of cameras with such software, also engendered anxiety associated with the feeling of having group participants intrude the private environment of individuals:

“I didn't like the idea of like bringing people I didn't know into my personal apartment and seeing my apartment, so I put it off for a long time, many months. I was coming to the Zoom meeting through the nurses. I trusted that avenue but with my support group…, I didn't attend until a number of months after I gave it a try and I actually started to… at first, I was very anxious” (Client 2).

Client 2 explained that the anxiety of having individuals gaze into their house environment caused them to boycott technology-mediated groups for a period. However, this anxiety was not transposable to situations where, in individual therapy, nurses gazed into participants’ environments. The relationship participants had with mental health nurses, which was infused with trust, seemed to act as a protective factor for such anxiety.

Anxiety experienced by participants was also rooted in some privacy concerns around the use of videoconferencing technologies for therapy. Some participants were worried about the confidentiality of the sensitive information exchanged via technological means. They believed in-person therapy was more secure for the exchange of personal health information:

“One of the things that concerns me, I'm not super secretive but a lot of sensitive information is talked about in therapy, and I know that there's all kinds of people listening to Internet conversations like through all these Snowden leaks and NSA. It's being recorded by everybody, everything that goes out on the internet so there's a lack of privacy. […] It's not as private, I find, as in person” (Client 1).

The CLWMI interviewed were cognizant of the confidentiality limitations associated with technology-mediated care. Despite these limitations, they still participated in technology-mediated therapy, while acknowledging they preferred the privacy of in-person meetings.

In a similar vein, participants explained that technology-mediated meetings had certain limitations associated with the social connectedness felt by individuals participating in the groups. The inability of videoconferencing software to create “social connectedness” was explained as the difficulty for conversations to flow inductively and cohesively, thus necessitating the presence of a group facilitator to moderate group dynamics:

“There's the social group, I haven't been to it yet, but I wonder how that would work without a facilitator to sort of guide how, when people spoke and didn't speak. I've had like social conversations like that after my support groups and sometimes we have difficulty in sort of everybody wants to talk at once kind of thing and it's hard to sort of decipher sometimes who's talking” (Client 2).

For others, the limits of videoconferencing regarding social connectedness materialized in a difficulty to read the nonverbal and social cues of other group participants:

“People don't interrupt each other as much [in person], like people on Zoom; it still happens in group settings, but people tend to speak at the same time and then you gotta say, “oh, I'm sorry” and let them speak. And maybe reading energy, reading social cues like nonverbal communication might be a bit better in-person I find…” (Client 1).

Many of our participants spoke about attending technology-mediated therapeutic groups about coping with social anxiety. Considering the social connectedness limitations associated with technology-mediated group delivery, one must wonder about the impact of this method of delivery on participants’ abilities to connect with others and cope with social anxiety.

### Role of nurses regarding transitioning in-person care to technology-mediated care

Transitioning nursing care from in-person to technology-mediated models was initially difficult for participants due to numerous factors, including accessing new technologies (hardware), adapting to them (software), and managing forced social isolation secondary to COVID-19 restrictions. To help participants through the difficulties associated with this transition, mental health nurses accompanied them every step of the way:

“I think it was over the phone is how [nurses] first contacted me and they just told me they're going to send a link. There's a lot of problems with… I guess I was a bit more savvy, so I had not very many problems and they didn't have to explain much to me. But there's other people in the groups, maybe a little more old [sic], they had problems. There was a lot of explaining to these people; guiding them to buy the right equipment; telling them how to use it… We would be on a Zoom call, and they'd have to phone them and explain things over the phone” (Client 1).

Beyond being professionals who provide group-based therapies related to social anxiety, mindfulness, self-compassion, substance use, counseling, and relaxation, mental health nurses were described as technological experts during the COVID-19 pandemic. They helped their patients access the hardware necessary to participate in virtual groups (computers, tablets, microphones, speakers, etc.) and taught them how to use the videoconferencing software (Zoom). Nurses were said to also provide real-time support to ensure individuals with technological difficulties are able to participate in group-based therapy:

“Now, if I have a problem with my tablet and I can’t get in or something's interfering with me getting in or whatever… I call [name of nurse] and she guides me through it, step by step, like she says, ‘what do you have on your screen’ and I tell her, and she says try this and try that… she's so patient” (Client 3).

## Discussion

For the CLWMI who participated in our study, the psychiatric hospital was more than a therapeutic space where care was dispensed; it was one of the rare places where they could meet, interact, socialize, and exchange with their peers and with hospital employees. Thus, when governmental restrictions were rapidly imposed to stop the spread of COVID-19 and outpatient mental health services were shut down, certain CLWMI lost more than their care; they lost their social networks. Such changes engendered fear and uncertainty, and resulted in increased anxiety, and isolation for our participants. To palliate this therapeutic and social isolation, mental health nurses transitioned the in-person care they provided to a virtual mode of delivery.

The CLWMI in our study identified various factors that modulated their experience of the in-person/virtual transition in mental health nursing care delivery. Factors that facilitated the transition included previous technological literacy and access to nurses who provided education and support about videoconferencing. Factors that complicated the transition included anxiety about technology, lack of access to appropriate hardware and software, and concerns about privacy. Despite these barriers, CLWMI in our study were overall satisfied with, and grateful for, the virtual care provided by mental health nurses, which is aligned with the results of other studies (Bhandari et al., 2011; Ellington, 2013; Naik et al., 2021).

Participants in our study had overall mixed feelings about technology mediated care. These feelings included gratitude for the interpersonal connection during the isolation of lockdowns, anxiety related to novelty of videoconferencing, and worry for their peers who were unable to access virtual services. These results are aligned with those of Trondsen et al. (2014) and Evans et al. (2017) to the extent that we did not find that the virtual delivery of nursing care negatively affected the nurse/patient therapeutic relationship. On the contrary, it seems to have strengthened existing therapeutic relationships and have initiated new ones for those CLWMI who were not already connected to mental health nursing services.

While some authors highlight the accessibility benefits of TMH care in a context of mental health specialist shortages (Southard et al., 2014; Trondsen et al., 2014), our results illustrated that virtual nursing care delivery may also enhance accessibility on an individual level. In effect, in line with the findings of Sugarman et al. (2021) and Wynn and Sherrod (2012), we found that the introduction of virtual nursing care made early morning groups accessible for participants who used medications with sedative effects, offered relief for participants who experience social anxiety, and eliminated the need for transit to the hospital. These benefits were, however, limited to those CLWMI who were able to access videoconferencing technology, leaving those without such access without care. On that note, we share Client 5's concern about the (absence of) care provided to persons who do not have access to appropriate hardware, software, or technological literacy in a future state where virtual nursing care provision may be the norm. Further, the in-person/virtual transition served as a catalyst for improved technological literacy in our participants. With this improved technological literacy, the CLWMI we interviewed were able to engage in virtual interpersonal connections in other social contexts (e.g., sending e-mails to family, sharing music with friends, etc.).

Underlying each patient's description of navigating virtual mental health care delivery is the identification of nurses as central actors in easing their transition. Patients identified several roles embodied by nurses, both traditional and supplemental to their nursing duties. Nurses were experts/teachers, personal information technology (IT) specialists, health care practitioners, counselors, and group facilitators. Nurses carried out these roles when providing therapeutic group therapies and one-on-one patient sessions. The CLWMI in our study expressed that the therapeutic relationship with nurses and their trust and attentiveness was paramount in successfully navigating the in-person/virtual transition. These results suggest that despite health care professionals’ concerns of virtual care negatively impacting the therapeutic relationship (Sugarman et al., 2021; Uscher-Pines et al., 2020), this was not the experience of CLWMI in our study. Technology-mediated care allowed CLWMI to independently manage their own mental health symptoms through acquiring or adapting skills to their new context during the COVID-19 pandemic.

### Strength and limitations

The main strength of our study is its novelty. It is the first time the transition of in-person nursing care to virtual nursing has been explored. We nevertheless highlight four limitations associated with our study. Firstly, in line with interpretative phenomenology principles, the purpose of the study was to capture and illustrate the variety and complexity of experiences related to the transition of mental health nursing care services from in-person modes of delivery to virtual ones. Therefore, our results are not generalizable to a whole population, nor should they be. Rather, our results can be transferrable to the extent that they can be leaned upon when hospital nursing administrators develop and implement perennial plans about nursing mental health care delivery in a post-pandemic context. Secondly, CLWMI who participated in our study were those who had access to virtual mental health nursing care services, which may have further excluded the voices of those CLWMI who were not able to obtain these services. We attempted to minimize this limitation by conducting in-person recruitment during nurse-patient clinical encounters. Thirdly, the participants ended up being recruited from the same outpatient program, thus limiting the breadth of experiences being studied. Finally, the demographic profile of our participants may be considered as a limitation. Our participants were mostly middle-aged White individuals with a high school-level education. Perspectives of Black, Indigenous, and other Persons of Color (BIPoC), of young adults, of elderly persons, and of people with a lower or higher level of education may therefore have been inadvertently silenced despite our inclusive recruitment approach.

### Recommendations

Our study is the only study that specifically explores the transition from in-person to virtual care delivery for mental health concerns. While other mental health studies looked at objective measures of effectiveness, such as changes to medication adherence and symptom management (Montes et al., 2010; Talarico, 2021; Uslu & Buldukoglu, 2020), we assessed the subjective experiences of service users, providing context for such measures of effectiveness.

Based on our findings, we propose three concrete recommendations. Firstly, nursing therapeutic groups should be offered both virtually and in-person. Beyond allowing for CLWMI to choose how they would like to attend their group; this has been identified by our participants as being essential for accessibility reasons. Indeed, offering virtual therapeutic groups allowed for CLWMI who ca not commute to the hospital to partake in care. This was especially relevant for our participants who took medications with sedative side effects. Secondly, groups targeting socialization should be held in-person. Participants explained that although the virtual offering of such groups was helpful during the COVID-19 pandemic context, videoconferencing technology caused significant barriers related to social connectedness, to the ability to read non-verbal cues and to the fluidity of verbal communication. For our last recommendation, hospital administrators should provide financial resources for clients to have the appropriate hardware and software to participate in therapeutic groups. Relatedly, hospital administrators should recognize the time commitment required for nurses to provide CLWMI with education about videoconferencing technology and with real-time assistance.

Rapid technological expansion occurred during the COVID-19 pandemic, which forced nurses to take on additional roles beyond what is traditionally considered nursing duties. Given that there are benefits to virtual care delivery, it is expected that use of technology in the delivery of patient care will continue to evolve. As such, nurses will need to be increasingly knowledgeable, adaptable, and innovative in using technology to provide client care. Education institutions preparing future nursing professionals must keep virtual care delivery models in mind when planning curriculum and clinical experiences to prepare nurses for modern nursing roles they will assume.

## Notes

1. Client 6 did not consent to have the interview recorded. Notes were taken during the interview. Therefore, the excerpts are written in the third person.
